# An immuno-lipidomic signature revealed by metabolomic and machine-learning approaches in labial salivary gland to diagnose primary Sjögren’s syndrome

**DOI:** 10.3389/fimmu.2023.1205616

**Published:** 2023-07-14

**Authors:** Geoffrey Urbanski, Floris Chabrun, Estelle Delattre, Carole Lacout, Brittany Davidson, Odile Blanchet, Juan Manuel Chao de la Barca, Gilles Simard, Christian Lavigne, Pascal Reynier

**Affiliations:** ^1^ Department of Internal Medicine and Clinical Immunology, University Hospital, Angers, France; ^2^ Mitolab, MitoVasc Institute, CNRS 6015, INSERM U1083, University of Angers, Angers, France; ^3^ Department of Orofacial Sciences, University of California, San Francisco, San Francisco, CA, United States; ^4^ Department of Biochemistry and Molecular Biology, University Hospital, Angers, France; ^5^ Centre de Ressources Biologiques, University Hospital, Angers, France

**Keywords:** kynurenine, biomarker, metabolomics, Sjögren’s syndrome, sicca, salivary glands, omics

## Abstract

**Introduction:**

Assessing labial salivary gland exocrinopathy is a cornerstone in primary Sjögren’s syndrome. Currently this relies on the histopathologic diagnosis of focal lymphocytic sialadenitis and computing a focus score by counting lym=phocyte foci. However, those lesions represent advanced stages of primary Sjögren’s syndrome, although earlier recognition of primary Sjögren’s syndrome and its effective treatment could prevent irreversible damage to labial salivary gland. This study aimed at finding early biomarkers of primary Sjögren’s syndrome in labial salivary gland combining metabolomics and machine-learning approaches.

**Methods:**

We used a standardized targeted metabolomic approach involving high performance liquid chromatography coupled with mass spectrometry among newly diagnosed primary Sjögren’s syndrome (n=40) and non- primary Sjögren’s syndrome sicca (n=40) participants in a prospective cohort. A metabolic signature predictive of primary Sjögren’s syndrome status was explored using linear (logistic regression with elastic-net regularization) and non-linear (random forests) machine learning architectures, after splitting the data set into training, validation, and test sets.

**Results:**

Among 126 metabolites accurately measured, we identified a discriminant signature composed of six metabolites with robust performances (ROC-AUC = 0.86) for predicting primary Sjögren’s syndrome status. This signature included the well-known immune-metabolite kynurenine and five phospholipids (LysoPC C28:0; PCaa C26:0; PCaaC30:2; PCae C30:1, and PCaeC30:2). It was split into two main components: the first including the phospholipids was related to the intensity of lymphocytic infiltrates in salivary glands, while the second represented by kynurenine was independently associated with the presence of anti-SSA antibodies in participant serum.

**Conclusion:**

Our results reveal an immuno-lipidomic signature in labial salivary gland that accurately distinguishes early primary Sjögren’s syndrome from other causes of sicca symptoms.

## 1 Introduction

Primary Sjögren’s syndrome (pSS) is an autoimmune disease characterized by the lymphocytic infiltration of exocrine glands (1). The demonstration of this exocrinopathy and its consequences on tear and saliva productions constitute most of the current items of the 2016 ACR/EULAR (American College of Rheumatology/European League against Rheumatisms) classification criteria (2). The evidence of exocrine glands involvement is based on a labial salivary glands (LSG) biopsy and the presence of focal lymphocytic sialadenitis (FLS) with calculation of the focus score (FS; number of clusters or foci containing ≥ 50 lymphocytes per 4 mm² of parenchyma) (3). LSG have the advantage of easy access compared to other exocrine glands and have been shown to be representative of major salivary glands (4). This identification of histological lesions is of major interest in patients without anti-SSA antibodies in order to certify the autoimmune character of the exocrinopathy. The LSG biopsy has good performances for pSS classification with sensitivity ranging from 79% to 91.4% and a specificity ranging from 81.8% to 100% (5–7). It also has a prognostic value with the presence of germinal centers and some lymphoid subpopulations being associated with systemic manifestations and a higher occurrence of lymphoma (8).

However, the diagnosis of FLS (with calculation of FS) does not take into account other lesions such as acinar atrophy, epithelial cells proliferation, or fibrosis (9), and is not appropriate for small focal infiltrates (10). Indeed, the LSG biopsy highlights the late histological consequences of exocrinopathy as the unstimulated salivary flow rate does for salivary function with a threshold of 0.1 mL/min (7). This contributes to the long delay in diagnosis often experienced by pSS patients (11). Consequently, there is a crucial need for better characterization of early stages of exocrine gland involvement to enable earlier diagnosis (12). In addition to various immune cells infiltrates (13), many chemokines in LSG have been associated with pSS status [chemokine C-C ligand 19 (CCL19) (14), CCL21 (14), CCL25 (15), chemokine ligand receptor (CCR7) (14), chemokine C-X-C ligand 1 (CXCL1) (16), CXCL9 (17), CXCL10 (15, 17), CXCL12 (14), CXCL13 (14), chemokine C-X-C ligand receptor 4 (CXCR4) (14), CXCR5 (14), CXCR1 (16), CXCR3 (17), lymphotoxins (LT) alpha and beta (14), Thymic Stromal Lymphopoietin (18)] and notably those associated with B lymphocytes development (19). However, few results have been reproduced except for BAFF (14, 20), CCL21 (14), and CXCL13 (14). In their study of salivary gland secretomes, Blokland et al. were able to correctly (95.8%) distinguish pSS from non-pSS sicca patients based on four chemokines, including CXCL13 (21). However, such parameters mainly focus on immune cells while the role of epithelial cells, which are of great importance in pSS pathogenesis (22), is poorly reflected. Omics approaches are unbiased and have opened new horizons in the study of SS pathogenesis. Studies based on RNA-Sequencing on LSG in pSS are scarce. However, two such studies have already highlighted the main role of the Inducible T cell costimulatory (ICOS) pathway (23). Proteomic studies on LSG essentially confirmed the implication of some already known proteins in pSS such as beta-2-microglobulin, lactoferrin, immunoglobulin kappa light chain, polymeric Ig receptor, lysozyme C and cystatin C, amylase and carbonic anhydrase VI (24), alpha-defensine-1 and calmoduline (25). However, some of these proteomic studies were unable to highlight any differences between samples from pSS and from non-pSS sicca patients (26).

Clinical metabolomics consist in measuring the concentration of a large number of metabolites in biological fluids or tissues and then modeling their predictive power for a pathological condition compared to a control group. Using this strategy, we have recently identified a metabolomic signature predictive of pSS in tears (27). To our knowledge, no study to date reported a metabolomic approach performed in LSG from pSS patients.

The present study aimed at i) identifying a metabolomic signature within LSG from participants with newly diagnosed pSS, compared to those presenting another non-immune cause of sicca symptoms; and ii) evaluating the variation in this metabolic signature with respect to age, sex, immunological status, and dryness severity.

## 2 Material and methods

### 2.1 Ethical considerations

The METABOGREN study was approved by the Ethical Committees of Angers University Hospital (CPP DC2014-2224, AC2017-2993, bioethics n°2018/42) and was conducted in compliance with the declaration of Helsinki. All participants signed a written informed consent.

### 2.2 Eligibility criteria

We included all incident patients with an objective measure of eye and/or mouth dryness between May 2017 and May 2018 in this study cohort, as previously described (27). Objective eye dryness was defined with a Schirmer I test ≤ 5 mm/5 min on at least one eye and/or an Ocular Staining Score (OSS) ≥ 3 and/or a break-up time (BUT) test < 10 seconds on at least one eye. Objective mouth dryness was defined as an unstimulated saliva flow (UWS) ≤ 1.5 mL/15 minutes. We assess all patients prospectively with respect to whether or not they met classification criteria for SS using the 2016 ACR-EULAR criteria. Cases were defined as those who met the 2016 ACR/EULAR criteria (2). Controls did not meet the 2016 ACR/EULAR criteria, had no anti-SSA or anti-SSB antibody, and had a LSG biopsy negative for FLS. Participants were enrolled using a consecutive sampling strategy until we had two groups with 40 participants each.

We excluded patients with a previous diagnosis of pSS and as follows: history of labial surgery; history of ocular surgery; wearers of contact lenses and scleral contact lenses; other systemic autoimmune disease (systemic lupus erythematosus, antiphospholipid syndrome, rheumatoid arthritis, immune-mediated myopathy, systemic scleroderma, mixed connective tissue disease, eosinophilic fasciitis, sarcoidosis, IgG4-related disease, ANCA-associated vasculitis, periarteritis nodosa, IgA vasculitis, Cogan’s syndrome, Behçet disease, Still disease); inflammatory eye disease (uveitis, ocular pemphigoid); graft versus host disease; solid cancer and/or lymphoma/leukemia without remission at least for 2 years except for MALT-associated B lymphoma of parotid; severe or non-controlled arterial hypertension; chronic heart failure, defined as left ventricular ejection fraction ≤ 30%; non-controlled diabetes defined as glycated hemoglobin ≥ 8% or diabetes with microangiopathy; chronic respiratory disease necessitating O_2_ support; chronic kidney failure defined as creatinine clearance with MDRD (Modified Diet for Renal Disease) formula ≤ 30 ml/min/1.73 m²; known mitochondria disease or metabolic genetic disorder; psychotic disorder or unstable psychiatric disease; use of systemic immunosuppressor or immunomodulatory drugs; use of systemic corticosteroids; and use of eye drops, with the exception of artificial tears. Pregnant women and patients unable to give informed consent were also excluded.

### 2.3 Data collection

Participants were instructed to discontinue using any artificial saliva or sialogogues 48 h before the biospecimen collection. All the following tests were performed in a standardized manner by the same operator: Schirmer I test (GECIS, Neung sur Beuvron, France), OSS for the lacrimal function, and UWS flow rate for the saliva production, were performed as previously described (7, 27). In addition, we collected the following data: age, sex, tobacco use, use of anticholinergic drugs (28), presence of autoantibodies (antinuclear antibodies, anti-SSA and anti-SSB antibodies, rheumatoid factors), presence of hypergammoglobulinemia (defined as gammoglobulin level over 1600 mg/dL), presence of cryoglobulinemia, and presence of extraglandular involvement of pSS (29).

### 2.4 Collection of labial salivary glands and metabolites extraction

Salivary glands were removed from the inferior labial mucosa in front of the canine teeth after local anesthesia with xylocaine. At least two glands were immediately placed in Eppendorf^®^ tubes (Eppendorf, Hamburg, Germany) into liquid nitrogen for transfer, and then stored at −80°C. Other glands were fixed in 4% paraformaldehyde, later transferred to 70% ethanol, then embedded in paraffin, and finally cut into 3 μm sections for histological staining. To determine the degree of lymphocytic infiltration, we used the focus score (FS < 1, FS = 1, FS > 1) and the staging according to Chisholm and Mason classification, as follows: no infiltrate (0), slight infiltrate (1), moderate infiltrate and FS < 1 (2), one focus (3) and more than one focus (4) (3, 30). The presence of granuloma and amyloid deposits was also systematically evaluated.

The cryopreserved LSG were firstly weighted for solvents normalization and then transferred in precooled 2.0 ml homogenization Precellys tubes (Bertin Technologies, Montigny-le-Bretonneux, France) prefilled with 1.4 mm diameter ceramic beads and microfiltered water. The glands were homogenized by two grinding cycles, each at 6500 rpm for 30 sec, spaced 20 sec apart, using a Precellys^®^ homogenizer kept at +4°C. The homogenate was centrifuged at 12,000 g for 5 min and 5 µL were separately collected for later protein quantification. Cold methanol (3 µL/mg) was added into the homogenate, followed by a new grinding cycle at 6500 rpm for 30 sec. Then, the supernatant was recovered after centrifuging the homogenate at 20,000 g for 10 min and kept at -80°C until metabolomic analysis (27).

### 2.5 Metabolite analysis

**Figure 1:**
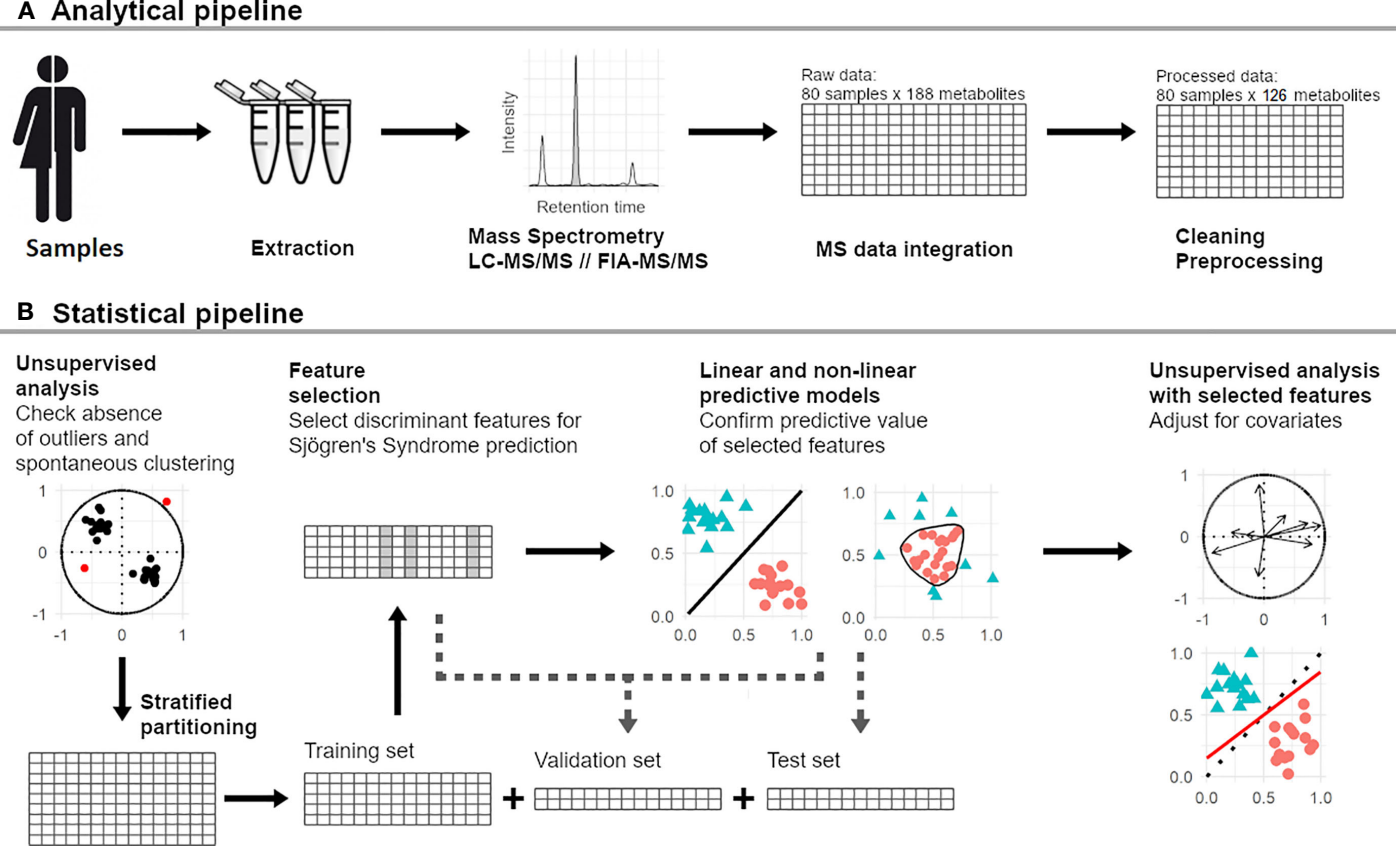
Analytical **(A)** and Statistical **(B)** workflow of the study. FIA, flow injection analysis; LC, liquid chromatography; MS, mass spectrometry; MS/MS, tandem mass spectrometry.

We applied a targeted, quantitative metabolomic approach to the tear extracts using the Biocrates^®^ AbsoluteIDQ p180 kit (Biocrates Life Sciences AG, Innsbruck, Austria) and an AB Sciex QTRAP 5500 (Life Sciences Holdings, Villebon-sur-Yvette, France) mass spectrometer (MS) as previously described (27) (**Figure 1A**). More details about the metabolomic kit, the MS and liquid chromatography (LC) parameters are available in **Supplementary Material**. All analyses were performed with the same kit to avoid batch effect.

### 2.6 Data cleaning and preprocessing

A cleaning step was performed to filter out unusable data: metabolites with more than 50% of values lying outside the quantification detection limits were removed from the datasets. For all potentially withdrawn features, a Chi-squared test for independence was performed between the number of values within bounds and out-of-bounds in pSS and non-pSS Sicca patients, to prevent removal of discriminating features, i.e. features with a significant difference between the 2 groups (31). Metabolite concentrations were normalized with protein concentration.

### 2.7 Statistical workflow

#### 2.7.1 General statistics

Continuous variables were summarized using medians, and 25^th^ and 75^th^ percentiles, and categorical variables with absolute values and percentages. For univariate analyses, we used Student t-test or Mann-Whitney test as appropriate for continuous variables, and Chi-squared test or Fisher’s test as appropriate for categorical variables. The type I error was 5%. Image manipulation and formatting were performed using GNU Image Manipulation Program (GIMP) software (version 2.10). The analyses were carried out using R software (version 4.0.1, R-project-org, Vienna, Austria) and SPSS (v23.0, IBM Corp, NY, USA). R packages are listed hereafter: FactoMineR for principal component analysis (PCA) (32); Rpart for stratified partitioning (33); glmnet for logistic regression with or without regularization (34) based on λ with 1 standard error (SE); randomForest (35); and pROC to evaluate the area under curves for receiver operating characteristic curves - area under the curve (ROC-AUC) (36).

#### 2.7.2 Determination of the metabolomic signature and influence of covariables

Full statistical workflow has been previously detailed (27) and is available in **Supplementary Material**. In brief, we randomly partitioned the study population into three data subsets with stratification on case-control status: training set (n = 52), validation set (n = 10) and test set (n = 18) (**Figure 1B**). To identify the best predictive model, we carried out a feature selection using elastic-net regularized logistic regression and we compared ROC-AUC from linear (elastic-net regularized logistic regression) and non-linear (random forests) machine learning architectures in the training and validation sets. The model with the highest performance was finally evaluated on the test set to determine the metabolomic signature. The main components of this signature were extracted by means of PCA and the influences of main confounders [age, sex, presence of anti-SSA antibody, tobacco use, use of anticholinergic drugs, rate of UWS test, and presence of lymphocytic sialadenitis and its grade (3)] were tested on them using linear regression.

## 3 Results

### 3.1 Study population characteristics

Among 100 patients explored for sicca symptoms, 90 were included in the cohort. The study population was composed of 40 patients who were found to meet classification criteria for pSS, and among whom 20 had anti-SSA antibodies, and 40 non-pSS Sicca patients randomly selected from the 50 remaining patients who met inclusion criteria as controls. Median age for the whole studied population was 60.5 [46.8-67] years with sex ratio (W/M) of 7.9. General characteristics are summarized in **Table 1**. The LSG weight and protein concentration did not differ between the two groups.

**Table 1:** General and immunological characteristics of the two groups, composed of pSS patients and non-pSS sicca patients.

	pSS (n=40)	Non-pSS Sicca (n=40)	p-value
Women n (%)	36 (90)	35 (87.5)	> 0.99
Age (years)	63 [51.8-67]	58.5 [46.8-66.3]	0.45
Tobacco use n (%)	17 (42.5%)	22 (55%)	0.37
Anticholinergic drugs use n (%)	17 (42.5%)	18 (45%)	> 0.99
Dry syndrome features			
Subjective dry mouth syndrome n (%)	36 (90%)	36 (90%)	> 0.99
Delay from first symptoms of mouth dryness to diagnosis (months)	39 [14-81.8]	32 [13.5-122]	0.60
UWS flow rate (1.5 mL/15 min)	3.2 [1.6-4.8]	4 [1.8-6.6]	0.12
Subjective dry eye syndrome n (%)	36 (90%)	35 (87.5%)	> 0.99
Delay from first symptoms of eye dryness to diagnosis (months)	42 [16.5-108.5]	42 [14-126.5]	0.66
Schirmer I test (mm/5 min)	2.3 [0-4.5]	3.25 [0.8-7.4]	0.03
Ocular staining score (highest value between both eyes)	4 [2-5]	2 [1-3]	< 0.0001
2016 ACR/EULAR criteria for pSS			
Schirmer I test ≤ 5 mm/5 min n (%)	39 (97.5%)	30 (75%)	0.007
OSS ≥ 5 n (%)	15 (37.5%)	5 (12.5%)	0.02
UWS flow rate ≤ 0.1 mL/min n (%)	10 (25%)	10 (25%)	> 0.99
Anti-SSA antibodies n (%)	20 (50%)	0	< 0.0001
Anti-SSB antibodies n (%)	12 (30%)	0	0.0002
Focus score ≥ 1 on LSG Biopsy n (%)	35 (87.5%)	0	< 0.0001
Immunological and clinical systemic involvement			
Antinuclear antibodies titer ≥ 1/320 n (%)	18 (45%)	5 (12.5%)	0.003
Gammoglobulin level over 1600 mg/dL n (%)	10 (25%)	1 (2.5%)	0.007
Presence of rheumatoid factors n (%)	15 (37.5%)	6 (15%)	0.04
Presence of cryoglobulinemia n (%)	3 (7.5%)	4 (10%)	> 0.99
Extra-glandular involvement of pSS n (%)	15 (37.5%)	–	
Characteristics of the labial salivary glands			
LSG weight (mg)	9.48 [5.41-15.88]	10.33 [6.03-13.71]	0.62
Protein concentration (mg/mL)	20.7 [11.5-29.5]	22.3 [15.6-29.6]	0.28

LSG, labial salivary gland; pSS, primary Sjögren’s syndrome; UWS unstimulated whole saliva. Continuous variables are presented as medians and 25^th^ and 75^th^ percentiles.

### 3.2 Metabolomic signature extracted from labial salivary gland tissue distinguishing pSS from non-pSS related causes of secretory hypofunction

Among the 188 measured metabolites, 62 were excluded as they were beyond the limits of detection. Unsupervised analysis did not reveal any spontaneous clustering nor outliers on principal component analysis. For the feature selection among the 126 metabolites, the logistic regression with elastic-net regularization isolated eight models with the best AUC (0.80) and, among them, seven highly consistent models (alpha coefficients: 0.10; 0.11; 0.15; 0.17; 0.21; 0.22; 0.37) with the same six metabolites of interest and one model (alpha = 0.09) with solely five metabolites but all included in the list of metabolites present in the seven other models. We selected the models including six metabolites, one biogenic amine and five phospholipids: kynurenine; lysophosphatidylcholines (LysoPC) C28:0; phoshatidylcholine diacyl (PCaa) C26:0; PCaaC30:2; phoshatidylcholine acyl alkyl (PCae) C30:1; and PCaeC30:2.

Subsequently, we sought the best learning architectures between linear and non-linear ones on the validation set. With the six previously selected metabolites, logistic regression yielded 23 models with AUCs of 0.84, homogenous with the prediction obtained on the training set (AUC of 0.80). The random forest disclosed AUCs of 0.72 whether over the six selected metabolites or the 126 metabolites, with a very large gap in comparison to the AUC of 1.00 obtained with random forest on the training set. Thus, we selected as the final model, the logistic regression with the lowest alpha value (alpha 0.30) to retain the maximum information among the metabolites of interest. The final prediction on the test set provided an AUC of 0.86. In the final signature, kynurenine was the sole metabolite with increased concentration in the pSS group compared to the non-pSS sicca group while all five phospholipids had decreased concentration.

### 3.3 Influence of covariates on the metabolomic signature

**Figure 2:**
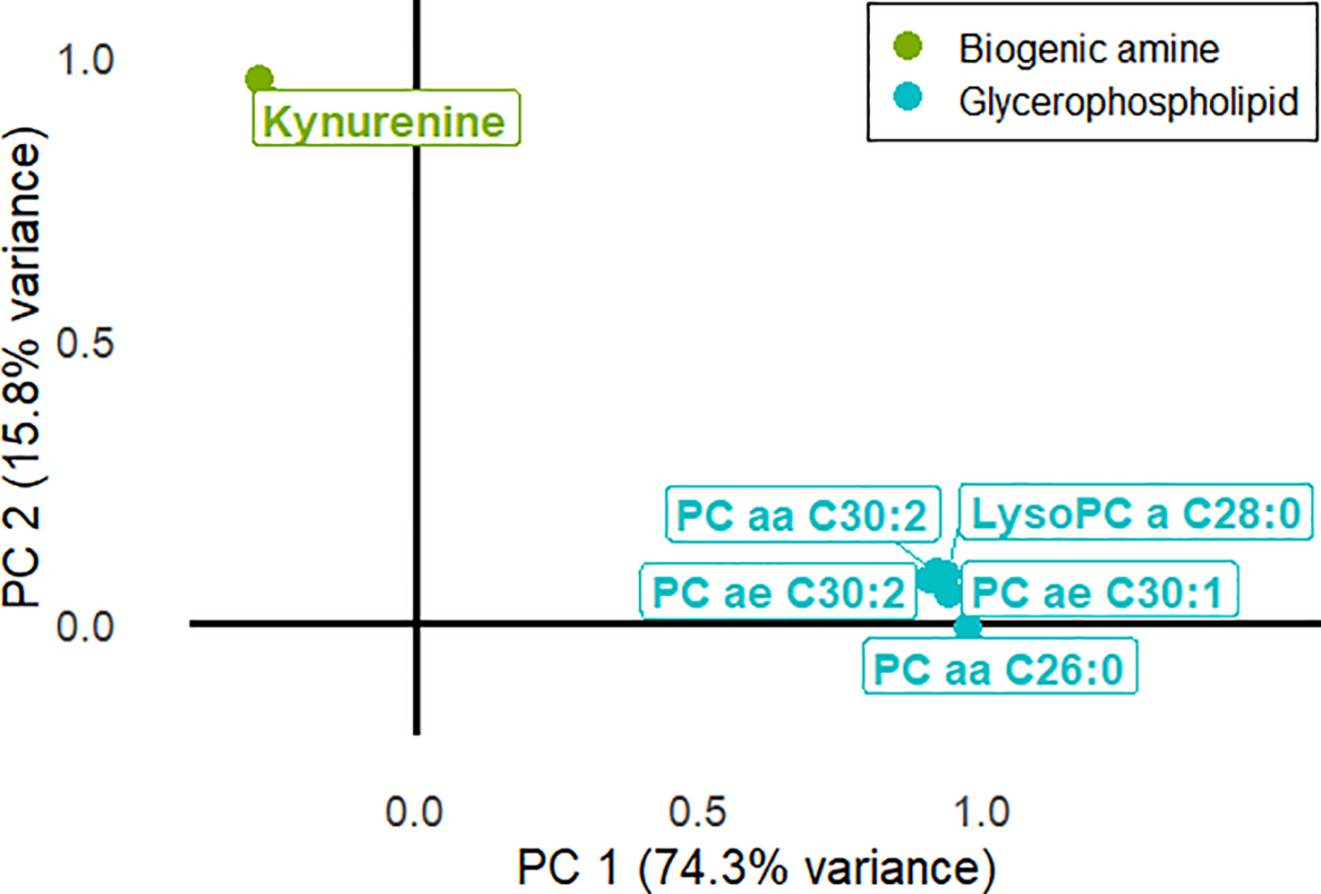
Distribution of the metabolites from the metabolomic signature in the two main components. the two main components were extracted after dimensional reduction with principal component analysis. LysoPC, lysophoshatidylcholine; PC, principal component; PCaa, phoshatidylcholine diacyl; PCae, phoshatidylcholine acyl alkyl.

Dimensional reduction of the metabolomic signature revealed that the two first principal components (PC) explained 91.1% of total variance (PC1 74.3%, PC2 15.8%). The distribution of metabolites over these two PC was clearly cut: the five phospholipids were mainly distributed in PC1 whereas the variance of kynurenine expressed itself in PC2 (**Figure 2**). Either gland weight or protein concentration were not significantly associated with the two main PCs: gland weight (PC1: p = 0.50; PC2: p = 0.55) and protein concentration (PC1: p = 0.70; PC2: p = 0.72) using linear regression models.

The association between the metabolomic signature and the pSS status was not altered by age, sex, tobacco use, use of anticholinergic drugs, and UWS flow rate in linear regression models analyzing the two first principal components (**Table 2**). The inclusion in these models of anti-SSA antibodies affected the significant association between the pSS status and the metabolomics signature for PC2 but not for PC1 (model 2). However, this association was disrupted for both PC1 and PC2 when including the lymphocytic sialadenitis grade (model 4). When excluding the pSS status and including both anti-SSA antibodies and lymphocytic sialadenitis grade in a new linear regression model, PC2 was strongly associated with the presence of anti-SSA antibodies (p = 0.002) but not with lymphocytic sialadenitis (p = 0.10). In comparison, PC1 was solely associated with the lymphocytic sialadenitis grade (p = 0.003).

**Table 2:** Influence of presence of anti-SSA antibodies and lymphocytic sialadenitis on the metabolomic signature.

*Model 1*	Principal Component 1 (mainly phospholipids)		Principal Component 2 (mainly kynurenine)	
	Beta	p-value	Beta	p-value
*pSS status*	-0.59	0.008	0.54	0.015
*Sex (male)*	0.11	0.75	-0.13	0.72
*Age*	-0.01	0.30	-0.02	0.11
*Tobacco use*	0.40	0.21	-0.16	0.61
*Anticholinergic drugs use*	0.37	0.11	-0.24	0.32
Model 2				
*pSS status*	-0.825	0.003	0.09	0.73
*Sex (male)*	0.12	0.74	-0.31	0.35
*Age*	-0.008	0.32	-0.01	0.08
*Anti-SSA antibodies*	0.35	0.25	0.95	0.001
Model 3				
*pSS status*	-0.58	0.01	0.48	0.03
*Sex (male)*	0.11	0.75	-0.13	0.71
*Age*	-0.009	0.39	-0.02	0.03
*Tobacco use*	0.39	0.23	-0.12	0.71
*Anticholinergic drugs use*	0.37	0.12	-0.24	0.31
*UWS flow rate*	0.02	0.68	-0.07	0.07
Model 4				
*pSS status*	-0.16	0.68	-0.04	0.91
*Sex (male)*	-0.05	0.90	0.08	0.82
*Age*	-0.009	0.36	-0.02	0.07
*Tobacco use*	0.24	0.48	0.05	0.88
*Anticholinergic drugs use*	0.26	0.29	-0.09	0.72
*Lymphocytic sialadenitis grade*	-0.18	0.16	0.25	0.06

The 4 models are independent. The value of Beta regressor is indicative for the direction of the association. Age, UWS rate, and grade of lymphocytic sialadenitis were quantitative data. Sex, presence of anti-SSA antibody, tobacco use, and use of anticholinergic drugs were dummy variables. pSS, primary Sjögren’s syndrome. UWS, unstimulated whole saliva.

## 4 Discussion

The metabolic signature we highlighted here is to our knowledge the first to be reported in the LSG in Sjögren’s syndrome. Its dichotomous composition, involving on one hand the increase of kynurenine known as one of the main immune-metabolites (37), and on the other hand the decrease of five phospholipids, leads us to consider it as an immuno-lipidomic signature. In addition, this dichotomy also reflects two distinct pathological phenomena since kynurenine was predominantly associated with the presence of anti-SSA antibodies and the phospholipid component with the intensity of lymphocytic infiltrates.

Concerning the five phospholipids (LysoPCaC28:0, PCaaC26:0, PCaaC30:2, PCaeC30:1, PCaeC30:2) with decreased concentration in pSS gland tissue, there is a reverse correlation between the phospholipid concentrations and the severity of lymphocytic sialadenitis (i.e., more intense was the grade more decreased were the concentrations). At first glance, this might suggest an inadequate production of phospholipids which could be consistent with previous works showing that a decreased concentration of phosphatidylcholines within dental plaque was associated with the risk of dental caries, including in pSS patients (38). Indeed, glycerophospholipids help to protect from caries by limiting diffusion of lactic acid (39). In addition, this is in accordance with findings from Tomita Y. et al. highlighting that saliva concentration of lipids correlates with salivary flow (40). Thus, this could link the intensity of sialadenitis, the reduced salivary flow, and the low saliva concentration of phospholipids to explain the severity of dental disorders in pSS. In addition, previous works noticed a significant increase of choline in saliva from pSS patients (41, 42), that could be related to this alteration of phosphatidylcholines concentration. The correlation between phosphatidylcholines impairment with the intensity of lymphocytic sialadenitis could be interesting for early diagnosis since these changes probably precede the occurrence of lymphocytes infiltrates, currently used in classification criteria, over the disease course.

The kynurenine is a biogenic amine synthesized from tryptophan under the action of indole-amine 2,3-dioxygenase (IDO), that can be transformed either into kynurenic acid *via* the kynurenine aminotransferase with properties of immunomodulation and neuroprotection, or into quinolinic acid *via* kynurenine 3-monooxygenase with proinflammatory and proapoptotic properties (43). The balance between those enzymes is regulated by the influence of different cytokines: type I and II interferons but also interleukine 1 (IL-1), IL-6, and TNF-alpha: IDO is finely regulated, its gene *Indo* containing a promoter stimulated by interferons (44), although IDO also induces biofeedback on interferon alpha through dendritic cells and CD80 (45). Imbalance of tryptophan/kynurenine has largely been documented in immune diseases (37) including pSS (46), in which increased activity of IDO (i.e., increased ratio kynurenine/tryptophan) was associated with an immunologically-active disease (46) and with intense interferon signature (44). Some authors emphasized the potential role of kynurenine in neurological involvement, chronic pain, and fatigue in pSS (47). To our knowledge, this study is the first to highlight the importance of kynurenine in salivary glands for pSS. In addition, Sardenberg W.M. et al. demonstrated that the elevation of plasma kynurenine was associated with greater adipose infiltration in LSG in pSS patients, suggesting that the pathway interferon-IDO-kynurenine could be implicated in salivary gland destruction (48). Our study brings a new milestone to those findings because we have unraveled a close relationship between the increase of kynurenine in salivary glands and the presence of anti-SSA antibodies in blood, even after adjustment for intensity of lymphocytic sialadenitis. Kynurenine might not only be a marker of gland destruction but the witness of an early specific immunological process within salivary glands in pSS, under the influence of type I interferons (23), that may precede the intense lymphocyte infiltrates. This would make kynurenine a candidate biomarker of high interest for early diagnosis of pSS.

The limitations of this study include the targeted metabolomic approach. Untargeted metabolomic studies would cover a wider part of metabolome. However, the untargeted approaches are also limited by their low level of standardization, with large variation of metabolite concentrations, which make them difficult to use in the discovery of clinical biomarkers. This study is also limited by its bulk nature, which did not allow us to precisely determine the source of metabolite changes within salivary glands, whether they originate from infiltrating lymphocytes or from epithelial cells. However, we were able to demonstrate that the phospholipidic component of our signature was correlated with the intensity of lymphocyte infiltration, unlike kynurenine. We hypothesized that alterations in kynurenine levels were more likely to be associated with qualitative changes related to the presence of systemic autoimmunity. In order to confirm this hypothesis and gain a deeper understanding of the underlying mechanisms, targeted analyses using techniques such as single-cell analysis, sorting, or microdissection will be necessary. However, the identification of kynurenine among the metabolic signature, a well-known metabolite for its role in immunometabolism balance, confirms the robustness of our findings and the potential for a clinical use.

To conclude, this study is the first to report a metabolomic signature able to distinguish pSS from non-pSS sicca syndromes at an early stage of the disease. The immuno-lipidomic signature has two main components, one with kynurenine is related to anti-SSA antibodies auto-immunity, while the phospholipid component is related to the intensity of lymphocytic infiltrates. These two components of the signature could therefore constitute excellent biomarker candidates for early diagnosis of pSS from the salivary glands, with the main objective of reducing the diagnostic errancy in this disease.

## Data availability statement

The raw data supporting the conclusions of this article will be made available by the authors, without undue reservation.

## Ethics statement

The studies involving human participants were reviewed and approved by Ethical Committees of Angers University Hospital (CPP DC2014-2224, AC2017-2993, bioethics n°2018/42). The patients/participants provided their written informed consent to participate in this study.

## Author contributions

GU, FC, CL and PR contributed to conception and design of the study. GU, ED, CaL, BD, JC, and GS collected and managed the samples and organized the database. OB managed the sample biocollection. GU and FC performed the statistical analysis. GU, FC, CL and PR wrote the first draft of the manuscript. All authors contributed to manuscript revision, read, and approved the submitted version.

## Glossary

**Table:**

ACR/EULAR 2016	American College of Rheumatology/European League against Rheumatisms
BUT	break-up time
CCL	chemokine C-C ligand
CCR	chemokine C-C ligand receptor
CXCL	chemokine C-X-C ligand
CXCR	C-X-C ligand receptor
FLS	focal lymphocytic sialadenitis
ICOS	inducible T cell costimulatory
IDO	indole-amine-2,3-dioxygenase
IL	interleukine
LC	liquid chromatography
LSG	labial salivary glands
LT	lymphotoxin
LysoPC	lysophosphatidylcholine
MALT	mucosis-associated lymphoid tissue
MDRD	modified diet for renal disease
MS	mass spectrometer
OSS	ocular staining score
PCA	principal component analysis
PCaa	phosphatidylcholine diacyl
PCae	phosphatidylcholine acyl alkyl
pSS	primary Sjögren’s syndrome
ROC-AUC	receiver operating characteristic curves - area under the curve
SE	standard error
TNF	tumor-necrosis factor
UWS	unstimulated whole saliva
