# Supplementary material for: An immuno-lipidomic signature revealed by metabolomic and machine-learning approaches in labial salivary gland to diagnose primary Sjögren’s syndrome
Source: Front Immunol. 2023 Jul 14;14:1205616. doi: 10.3389/fimmu.2023.1205616 (PMC10375713; doi:10.3389/fimmu.2023.1205616)
Supplement: Supplementary file 1 [file DataSheet_1.docx]

**An immuno-lipidomic signature revealed by metabolomic and machine learning approaches in labial salivary gland to diagnose primary Sjögren's syndrome**

**Short title: Immuno-lipidomics of salivary glands in Sjögren’s syndrome**

Geoffrey Urbanski^1,2,3 *^, Floris Chabrun^2,4^, Estelle Delattre^1^, Carole Lacout^1^, Brittany Davidson^3^, Odile Blanchet^5^, Juan Manuel Chao de la Barca^2,4^, Gilles Simard^2,4^, Christian Lavigne^1* †^, Pascal Reynier^2,4 †^

^§^ Contributed equally to this work and share last authorship

^1^ Department of Internal Medicine and Clinical Immunology, University Hospital, Angers, France

^2^ Mitolab, MitoVasc Institute, CNRS 6015, INSERM U1083, University of Angers, France

^3^ Department of Orofacial Sciences, University of California, San Francisco, CA, USA

^4^ Department of Biochemistry and Molecular Biology, University Hospital, Angers, France

^5^ Centre de Ressources Biologiques, BB-0033-00038, University Hospital, Angers, France

*** Corresponding author**: Geoffrey Urbanski, MD, MPH, PhD. Department of Internal Medicine and Clinical Immunology, 4 rue Larrey, 49000 Angers, France / Mitolab Team, MitoVasc Institute, CNRS 6015, INSERM U1083, University of Angers, 4 rue Larrey, 49000 Angers, France / Department of Orofacial Sciences, University of California, 513 Parnassus Ave, San Francisco, CA 94143, USA ; Phone: +33 2 41 35 40 03 ; Fax: +33 2 41 35 49 69 ; Email: [urbanskigeoffrey@gmail.com](mailto:urbanskigeoffrey@gmail.com)

**Supplemental material**

**Metabolite analysis**

***Analytical pipeline***

Targeted quantitative metabolomics analysis was carried out using the Biocrates® Absolute IDQ p180 kit (Biocrates Life sciences AG, Innsbruck, Austria) coupled with a QTRAP 5500 (SCIEX, Villebon-sur-Yvette, France) mass spectrometer (MS), a 1260 Agilent® High Performance Liquid Chromatography (HPLC) system and an ECLIPSE® XDB-C18 3.5μm 3.0 x 100 mm column (Agilent Technologies, Santa Clara, CA, USA). All reagents used in this analysis were of LC-MS grade and purchased from VWR (Fontenay-sous-Bois, France) and Merck (Molsheim, France). The metabolomics kit enables the quantification of up to 188 different endogenous molecules, including acylcarnitines (40), amino acids (21), biogenic amines (21), glycerophospholipids (90), sphingolipids (15) and hexoses (1). Flow-injection analysis (FIA-MS/MS) was used to quantify acylcarnitines, glycerophospholipids, sphingolipids and sugar, whereas liquid chromatography allowed us to separate amino acids and biogenic amines prior to detection with mass spectrometry (LC-MS/MS). Samples were prepared according to the Biocrates Kit User Manual. Briefly, extracts were vortexed thoroughly for 5 min then 10 μL were mixed with isotope-labeled internal standards and samples were loaded onto the center of the filter placed on the upper wall of the well in a 96-well plate. Metabolites were extracted in a methanol solution using ammonium acetate after drying the filter spot under a nitrogen flow and derivatizing with phenylisothiocyanate for the quantification of amino acids and biogenic amines. Extracts were diluted with MS running solvent (MilliQ water for HPLC assay or a methanol solution for FIA assay) prior to FIA and LC-MS/MS analyses. Peaks were integrated to retrieve raw data as a matrix compiling the concentrations of the 188 metabolites for the 80 samples, in addition to the calibration samples and quality control (QC) values. Three QCs composed of three concentrations of human samples − i.e., low (QC1), medium (QC2) and high (QC3) − were used to evaluate the performance of the analytical assay. A seven-point serial dilution of calibrators was used to generate calibration curves. Values of the coefficient of variation (CV = standard deviation/mean × 100, in %) associated with QC samples were used to validate quantitation in samples (CV threshold of 30 %). The software Analyst (SCIEX, Villebon-sur-Yvette, France) was used for MS data collection and the software MetIDQ® (Biocrates Life sciences AG, Innsbruck, Austria) was used to monitor the entire assay workflow.

***Mass Spectrometry parameters***

**1. Negative polarity Flow Injection Analysis (FIA-MS/MS)**

Scan Type: MRM

Ion Source: Turbo Spray

CUR: 20.00

CAD: Medium

IS: -4500.00

TEM: 200.00

GS1: 40.00

GS2: 50.00

DP: -55.00

EP: -10.00

CE: -12.00

CXP: -15.00

**2. Positive polarity Flow Injection Analysis (FIA-MS/MS)**

Scan Type: MRM

Ion Source: Turbo Spray

CUR: 20.00

CAD: Medium

IS: 5500.00

TEM: 200.00

GS1: 40.00

GS2: 50.00

EP: 10.00

CXP: 15.00

**3. Positive polarity Liquid Chromatography (LC-MS/MS)**

Scan Type: Scheduled MRM

Ion Source: Turbo Spray

CUR: 20.00

CAD: Medium

IS: 5500.00

TEM: 500.00

GS1: 40.00

GS2: 50.00

EP: 10.00

CXP: 15.00

**Abbreviations:**

MRM: Multiple Reaction Monitoring, CUR: Curtain gas flow rate, CAD: Collision gas, IS: IonSpray Voltage, TEM: Temperature of ion source, GS1: Ion Source Gas 1 (nebulizing gas) flow rate, GS2: Ion Source Gas 2 (drying gas) flow rate, DP: Declustering Potential, EP: Entrance Potential, CE: Collision Energy, CXP: Collision cell Exit Potential

***Liquid chromatography parameters***

Injection volume: 10 μL

Column temperature: 50 ± 1°C

| **Total time (min)** | **Flow Rate (µL/min)** | **Phase A^1^ (%)** | **Phase B^2^ (%)** |
| --- | --- | --- | --- |
| 0.00 | 500 | 100.0 | 0.0 |
| 0.50 | 500 | 100.0 | 0.0 |
| 5.50 | 500 | 5.0 | 95.0 |
| 6.50 | 500 | 5.0 | 95.0 |
| 7.00 | 500 | 100.0 | 0.0 |
| 9.50 | 500 | 100.0 | 0.0 |

^1^Phase A: formic acid/water (0.2% v/v)

^2^Phase B: formic acid/acetonitrile (0.2% v/v)

**Supplemental figure**: Representative LC-MS/MS spectra obtained by the p180 kit


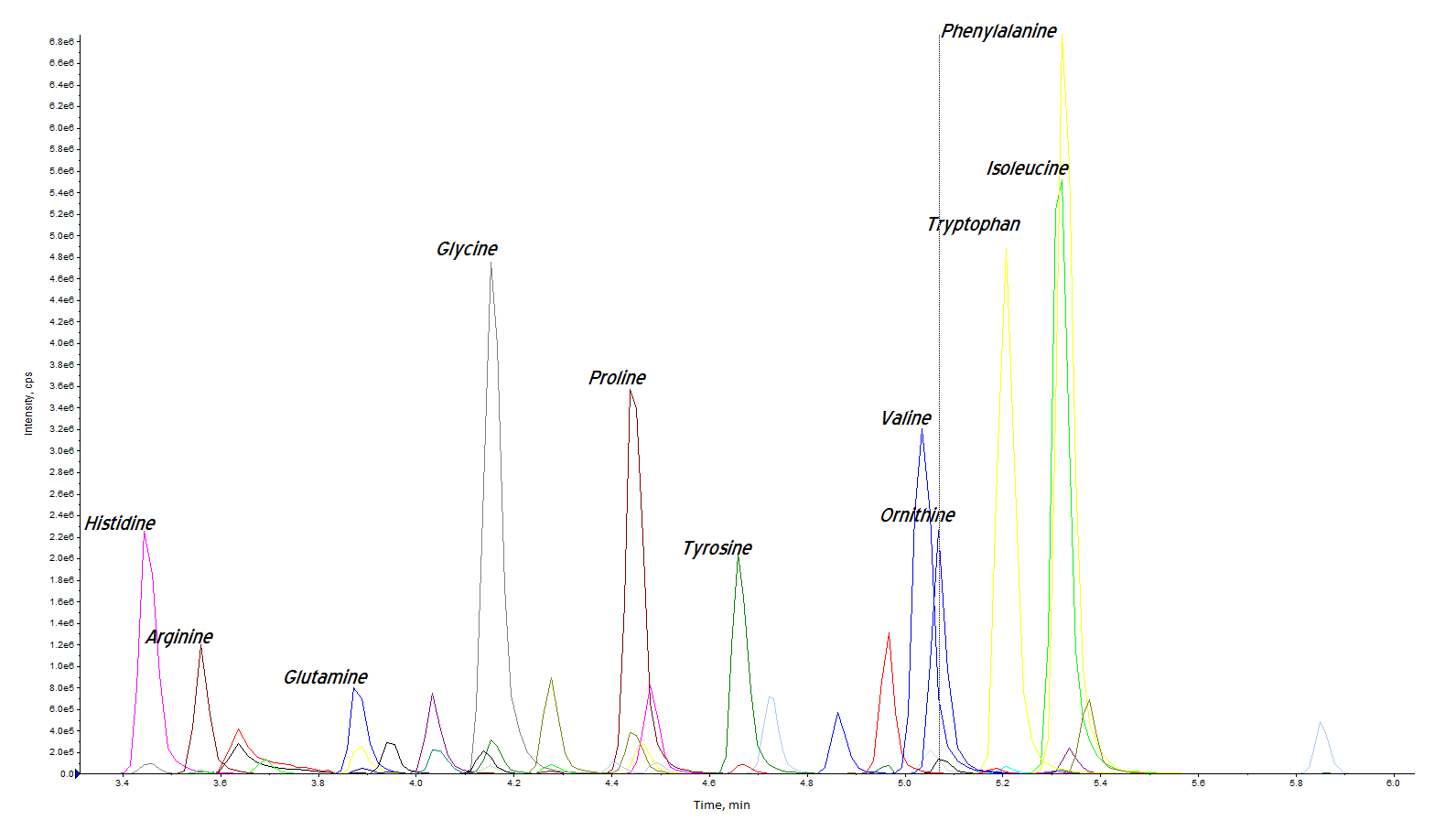


**Detailed statistical pipeline**

***Feature selection***

First, we performed unsupervised analysis using PCA to search for spontaneous group clustering and major outliers. We randomly partitioned the study population into three subsets with stratification on age, sex, and case-control status. We first extracted a test set (n = 18) with 9 pSS and 9 non-pSS participants, then a validation set (n = 10) with 5 cases and 5 controls and created the training set with the remaining 26 pSS and 26 non-pSS participants. The feature selection was made using elastic-net regularized logistic regression. A grid search was performed for values of parameter α for elastic-net regularization between 0 and 1, with a step of 0.01. For each model, optimal λ was determined by 10-fold cross-validation. After training, all models were ordered according to the receiver operating characteristic – area under the curve (ROC-AUC) of their predictions on the validation set. The optimal number of features was determined by selecting the model with the highest ROC-AUC sharing with non-zero coefficient for the same variables as the largest number of models with the same ROC-AUC.

***Model training and determination of a metabolomics signature***

Grid search was used to compare linear and non-linear machine learning architectures: elastic-net regularized logistic regression with α values ranging from 0 to 0.99 (α = 1 was excluded since feature selection had been performed previously) and random forests. Random forests were also trained with all metabolites during this step to check that metabolites excluded during the features selection step retained no discriminant information useful for the prediction (i.e., to check the absence of increase in performance when including feeding the excluded metabolites). Models were compared by their ROC-AUC on the validation set. Comparison was based on λ with 1 standard error (SE). The model with the highest performance was finally evaluated on the test set. Results on all datasets were compared to check the robustness and capability of generalization of the final model. The importance of the variables of interest was summarized by means of their regressor coefficients for logistic regression or mean Gini index for random forests.

***Influence of covariates on the metabolomic signature***

First, we summarized the variables from the metabolomic signature to the main principal components, obtained by means of principal component analysis. The influence upon the metabolomic signature of age, sex, presence of anti-SSA antibody, tobacco use, use of anticholinergic drugs, rate of UWS test, and presence of lymphocytic sialadenitis and its grade^1^ was evaluated by means of linear regression. Age, UWS rate, and grade of lymphocytic sialadenitis were quantitative data. Sex, presence of anti-SSA antibody, tobacco use, and use of anticholinergic drugs were dummy variables. The assumptions for linear regression were checked as follows: the linearity was evaluated graphically on scatter plots; the independence of the residues was checked with the Durbin-Watson test; and the distribution of the residues was appreciated graphically on QQ plots and on residuals vs fitted plots.

**References for Supplementary Material**

1. Chisholm, D. M. & Mason, D. K. Labial salivary gland biopsy in Sjögren’s disease. J Clin Pathol 21, 656–660 (1968).
